# Insinuating electronics in the brain

**DOI:** 10.1016/j.surge.2016.03.003

**Published:** 2016-08

**Authors:** Mark A. Hughes

**Affiliations:** Clinical Lecturer and Specialist Trainee in Neurosurgery, University of Edinburgh Centre for Clinical Brain Sciences and Department of Clinical Neurosciences, Western General Hospital, Crewe Road South, Edinburgh, EH4 2XU, United Kingdom

**Keywords:** Neurosurgery, Electronics, Micro-electromechanical system, Sensor

## Abstract

There is an expanding interface between electronic engineering and neurosurgery. Rapid advances in microelectronics and materials science, driven largely by consumer demand, are inspiring and accelerating development of a new generation of diagnostic, therapeutic, and prosthetic devices for implantation in the nervous system. This paper reviews some of the basic science underpinning their development and outlines some opportunities and challenges for their use in neurosurgery.

## Introduction

**Fig. 1:**
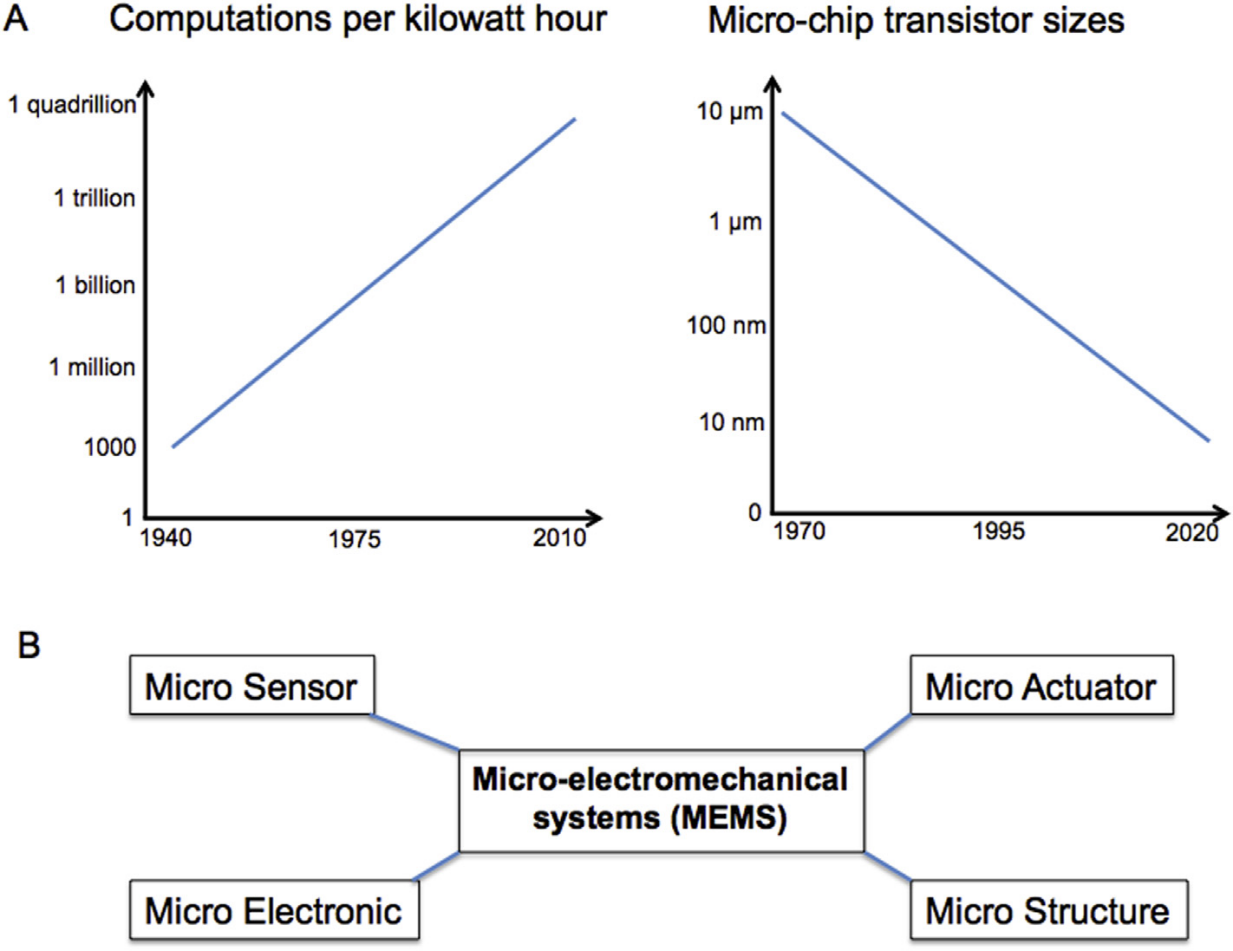
(a) Trends showing the rapid and persisting increase in computational power, and decrease in microchip size, in recent decades (based on data from Refs. 2, 3). (b) The component parts of the archetypal micro-electromechanical system.

Charles Babbage pioneered early mechanical computing devices in the 1820s.^1^ Today's computers have a predominantly microelectronic substrate and their performance, efficiency, and affordability continue to improve rapidly and predictably^2^, ^3^ (see Fig. 1A). By the 1980s, this allowed development of *portable* electronic devices. Now even smaller and more energy-efficient microelectronic devices are enabling the transition from portable to wearable to *implantable*. In tandem with an improving understanding of neuro–biotic interfaces and the computational machinery of the brain, such advances are enabling new ways to invasively monitor, interact, and intervene with nervous systems.

Micro-electromechanical systems (MEMS) combine miniaturized mechanical and electromechanical elements.^4^ Their physical dimensions range from several millimetres to well below one micron. The functional elements of MEMS are shown in Fig. 1B. MEMS transduction components (microsensors and microactuators) convert energy from one form to another and have particular relevance in biomedical applications. A wide range of microsensors now exist, including those that measure temperature, pressure, magnetic fields, radiation, impedance, inertial forces, and different chemical species. Micro-actuators include tools capable of ablating tissue (using heat, light, or ultrasound, for example) and tools for controlled delivery of bioactive molecules (such as chemotherapy or neurotransmitters). Others include micro-valves to control fluid flow, optical switches to modulate or redirect light, and micro-resonators.

The production methods used for MEMS mirror those used for batch fabrication in the integrated circuit industry. Once production reaches scale, this serves to lower production costs and increase reliability and functionality. MEMS (and their nano-scale equivalent, NEMS) enable the development of complete systems-on-a-chip: sensors collect information that is processed locally and used to direct actuators that alter aspects of the surrounding environment. In an implanted *in vivo* context, this model has numerous potential applications.

Usefully, the nervous system itself is governed by electronic signals: ions in solution move through membrane-bound channels in neurons, whilst electrons move within the solid-state lattices of microelectronic semiconductors. Hybridising the two systems to create a neuro–bionic interface is therefore a logical proposition, though one with multiple biological and engineering challenges. Beyond offering new ways of monitoring and intervening, hybrid systems can link neurons to prosthetic effectors; thereby offering a means of restoring function by circumventing an area of nervous system damage. This addresses the nervous system's very restricted capacity to recover or reorganise, and may finally allow neurosurgeons to mitigate *primary* brain injury. This paper outlines some of the challenges and opportunities for CNS-implanted MEMS.

## Challenges

The CNS is an unforgiving environment in which to intervene at all, let alone implant electrical devices. Complex neuroanatomy on a relatively small scale, notable vascularity, and conspicuous fragility are all challenges to implantation. Beyond these pragmatic surgical considerations, a fundamental challenge for all bionic systems is the interface between living tissue and implanted material.^5^ The host response to implantation of a foreign body tends to result in encapsulation. In the brain this takes the form of gliosis, resulting in insulation of the electrode or implanted component.^6^ Ideally, implanted systems would induce minimal foreign body response, allowing an intimate, long-term interaction with specific cells (or even subcellular components). These challenges have spurned extensive materials science and electrical engineering research that aims to engineer a sympathetic interaction and long-term functional connection between neurons and microelectronic systems.

For neuro-prosthetic devices, there is also the pre-requisite to interface with the *computational* apparatus of the brain. This is a massive challenge. The human brain contains ∼86 billion neurons, each with ∼7000 synapses, cooperatively performing ∼12 × 10^15^ computations per second.^a^ Different neurotransmitter types, the variable influence of glial cells, and a dynamic ultrastructure complicates the situation further. Moreover, neuronal organisation and connectivity evolve during development, ageing, and in response to pathology.

**Fig. 2:**
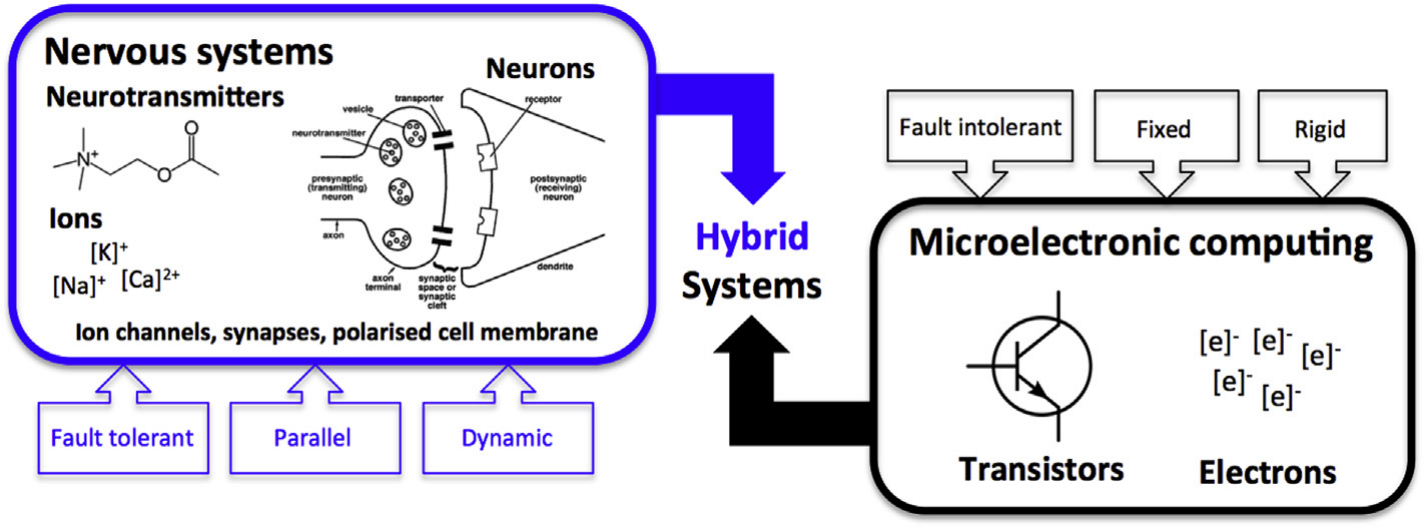
A comparison between the components and modi operandi of nervous systems and silicon-based computers (reproduced from Ref. [7]).

Whilst electronic signalling is central to both domains, there remain fundamental differences in computational strategy.^7^ Most microelectronic platforms operate in a sequential, rigid, and fault intolerant mode. In contrast, nervous systems comprise dynamic interconnected neurons with an intrinsic fault tolerance (see Fig. 2).

Beyond these biological and engineering issues, there are ethical considerations when intervening with the brain. Its complexity, coupled with its significance in human existence, demands strong justifications for interventions of this sort. Maintaining autonomy and protecting privacy is key, and relate directly to maintaining trust during development and deployment of novel neurotechnologies.^8^

## Experimental approaches to interaction

Intra-cortical implantation of electrodes, or any MEMS component, is highly invasive. There is inevitable parenchymal damage together with risk of bleeding, infection or seizure. Foreign bodies induce activation and migration of microglia and astrocytes. Reactive gliosis around electrodes impedes electrical conduction, as well as causing some local neuronal cell death. Improving our understanding of the abiotic:biotic interface is key. Much of the basic science work in this area involves efforts to hybridise microelectronics with simple neuronal networks *in vitro*; a pragmatic environment in which to hone technologies. One of the core challenges is to engineer a long-term sympathetic connection between the key processing components of neurons (ion channels) and those of electronics (electrodes and transistors). Several groups approach this challenge by trying to gain topographic control of the neuron or neurite (in an environment promoting long-term survival) and using this to guide its engagement with electrodes.^9^, ^10^, ^11^, ^12^, ^13^, ^14^

Techniques tested *in vitro* include the use of microcontact printing, where a microscopic stamp is used to print pro-adhesive proteins (such as vitronectin or fibronectin) onto a given surface (often silicon wafers) to define specific cell adhesion.^15^ Similarly, inkjet printers have been used to pattern pro-adhesive substances onto otherwise cytophobic backgrounds.^16^ This method has enabled rat hippocampal neurons and glia to be patterned successfully. Modifying surface roughness or other topographic characteristics can also be used to inform neuronal adhesion.^17^ Some of these techniques lend themselves to use with multi-electrode arrays (MEAs). For example, Marconi et al. aligned microcontact printing (using a silicon master) with a multi-channel MEA, to both control location of hippocampal neurons and also record electrophysiological characteristics.^18^ Similarly, Boehler et al. aligned a polymeric silicon-based stamp (‘inked’ with polylysine) with a MEA-incorporated substrate.^19^ The underlying electrodes recorded spike activity from specific parts of the neuronal network.

Beyond interfacing with specific neuronal components, better strategies are needed to maintain a long-term and reliable contact between the lipid bilayer of the polarised neuron and the oxide layer of silicon. Key variables are resistance and distance. Novel electrode designs are one way of improving and maintaining this contact. Carbon nanotubes (CNTs) are electrically conducting and have excellent interfacial electrical impedance.^20^ Sorkin et al. has cultured neurons on 20 μm CNT islands on a background of quartz.^21^ Neurons entwine and anchor themselves to these CNT islands, promoting a high fidelity electrical interface. Another approach involves altering the nature of the electrode:neuron interface by delivering bioactive molecules during or after implantation. For example, neurotrophic factors (to facilitate neurite outgrowth and neural preservation) or anti-inflammatory drugs can be delivered adjacent to the electrode or implanted device.^22^, ^23^ These technologies all build towards sympathetic, minimally disruptive, high channel, sub-cellular resolution MEMS implantation tools.

## Clinical applications and opportunities

Implanted electronic systems are already well-established in some neurosurgical settings (e.g. deep brain stimulation and vagus nerve stimulation) and deployed experimentally in others (e.g. invasive neuroprosthetic devices). As relevant technology matures, applications are expanding.

## Sensors

**Fig. 3:**
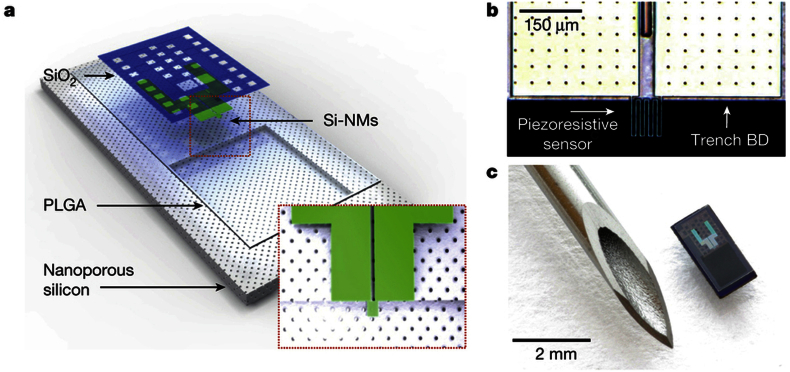
Adapted from Ref. [24] (a) Schematic illustration of biodegradable pressure sensor. Inset shows location of the silicon-nanomembrane strain-gauge. (b) Optical micrograph of the strain-gauge region. (c) Image of complete device. The outer diameter of the hypodermic needle is 1 mm.

Multi-modality sensors of intracranial pressure, temperature and brain oxygen saturation are established tools in well-resourced neuro-intensive care units. Most commonly a single, temporary, *wired* transducer array is placed via burr-hole, with real time measurements used to optimise physiological parameters such as cerebral perfusion pressure and brain oxygenation, and to guide the need for interval CT scan or surgical intervention. These devices have changed very little in recent decades and use old technology. Next generation MEMS sensors have the potential to hugely expand this approach. For example, Kang et al. have recently developed a miniaturised bio-resorbable nano-porous silicon sensor of temperature and pressure with dimensions of just 1 mm × 2 mm × 0.08 mm^24^ (see Fig. 3). *In vivo* tests of intracranial pressure in rat brain compared well with existing techniques. Uniquely, the sensor itself dissolves over time when exposed to biofluids (such as cerebrospinal fluid), leaving only biocompatible end products. It is also amenable to *wireless* transmission of information.

In the management of glioma, implanted sensor arrays embedded in the resection cavity may enable early detection of tumour recurrence, rather than via interval MRI as occurs at present. Such arrays could detect changes in tissue impedance, hypoxia, pH, or temperature to characterise and identify the hallmarks of tumour progression. Such early warning systems would allow proactive rather than reactive deployment of secondary therapies, and might also help to differentiate true tumour progression from radio-necrosis (a well-described problem in neuro-oncology). Moreover, combining the sensor array with a MEMS component capable of lesioning adjacent tissue would allow immediate *in situ* treatment. A locally deployed therapy (e.g. hyperthermia induced by passing a current between two electrodes, or ultrasound, or UV light, or release of an aliquot of chemotherapy) may have a better side-effect protocol than systemically administered therapies whose tissue penetrance is also restricted by the blood brain barrier.

MEMS-based sensors also have a role in improving the management of hydrocephalus. The primary treatment for hydrocephalus is still a cerebrospinal fluid shunt (usually draining to the peritoneal cavity). Whilst life saving, shunts have high failure rates and are fundamentally crude devices. Whilst variable flow and variable pressure valves have been developed, there is a need for systems capable of delivering more advanced control, feedback, and communication. A ‘smart shunt’ of this sort has been envisaged for decades.^25^ Reliable sensors in shunts could relay information about shunt functionality, CSF pressures, and the presence of infection. Similarly, MEMS sensors may have a role in the management of degenerative spinal conditions. Sensors measuring pressure or acceleratory forces may guide development of intelligent implants, capable of ameliorating against adjacent level disease or pull-out of pedicle screws, for example. For all of these systems, where internal variables are transduced in real or near real time, robust and secure systems are needed to communicate and integrate data. With wireless transmission to internet-connected smartphones, such sensors become part of the ‘internet of things’.

## Stimulators

Stimulation of the brain, spine, and peripheral nervous system is a well-established aspect of contemporary ‘functional’ neurosurgery. Deep brain stimulation (DBS) has a good evidence base in Parkinson's disease,^26^ essential tremor and dystonia,^27^ and refractory chronic pain syndromes.^28^ Its use in other contexts (e.g. refractory depression, obsessive compulsive disorder, epilepsy, eating disorders, addiction, cognitive decline) is under investigation. Peripheral nervous system stimulators (such as occipital and vagal nerve stimulators) are also in routine clinical use.

Crucially, current devices are somewhat crude in their interaction; stimulating relatively large regions of tissue indiscriminately. In DBS for Parkinson's disease, for example, this results in unwanted cognitive and emotional side effects. In tandem with improved understanding of disease-specific neural circuits, advanced MEMS devices offer means of stimulating the nervous system with more specificity and delicacy. For example, early iterations of DBS hardware are now making way for smaller, more complex and more sophisticated electrodes capable of better-targeted stimulation.^29^, ^30^

The growing field of *in vivo* piezo-electric energy scavenging (whereby movement of body tissues is used to generate electricity) also offers a route to meeting the long-term energy requirements of implanted electrical devices, without the need for batteries.^31^ As these opportunities present themselves, so interest from industry also grows.^32^

## Invasive neuroprostheses

A neuroprosthesis creates a *de novo* connection between the nervous system and the external world, mediated by an intervening computer. By incorporating a prosthetic ‘effector’, the new connection can enable a behaviour. Simple *input* neuroprosthetic devices have existed for decades, most notably the cochlear implant which uses electronics to transduce sound and, in near real time, stimulate the cochlear nerve. Pathology affecting any CNS component downstream of cortex (or any CNS-innervated structure) is theoretically amenable to this form of therapy. Electrical activity is recorded from functioning cortical regions (e.g. motor cortex), then decoded in near real time, and used to control the effector (e.g. robotic arm). This allows an area of pathology (e.g. spinal cord injury) to be circumvented and a functional interaction with the outside world re-established. The extent to which a new activity can deliver real world benefit depends upon both the underlying pathology and the fidelity of the neuroprosthesis itself. Current electrode grids used to record from cortex can record and ‘translate’ only a small proportion of cortical activity. As devices become smaller and interact at higher resolution, neuroprostheses will become higher fidelity. Current prosthetic devices range from an electric wheelchair, to an innervated robotic limb,^33^ to a synthetic exoskeleton, to an artificial sphincter. However, the effector or prosthesis need not necessarily interact with the *tangible* world. Rather, it may exist in an online virtual world manifesting as an electronic avatar. Human BCI control of an on-screen computer cursor illustrates feasibility of this concept.^34^

## Conclusion

New therapeutic opportunities are arising due to advances in both microelectronics and neurobiology. Translating these advances into new therapies is challenging and will demand innovative collaborations amongst engineers, biologists, surgeons, and industry.

## Conflicts of interest

none.

## Footnotes

a This approximation is based on assumptions of 86 billion neurons, connected via 7000 synapses per neuron, firing at an average frequency of 20 Hz, resulting in 1.204 × 10^16^ firing events per second. Moreover, this approximation fails to appreciate other “calculations” attributable to glia:neuron interactions or neuropeptides, for example.
